# Multimodal hypersensitivity across the menarcheal transition is associated with pelvic pain in adolescents: a longitudinal study

**DOI:** 10.64898/2026.09.09.26362449

**Published:** 2026-09-11

**Authors:** Matthew J Kmiecik, Kevin M Hellman, Sarah E Darnell, Gregory E Miller, Frank F Tu

## Abstract

Multimodal hypersensitivity (MMH), heightened sensitivity across multiple sensory modalities, is associated with chronic pain risk in adults. How MMH develops across adolescence and whether early pain experiences shape its trajectory remains unknown. The Early Menstrual Pain Impact on Multisensory Hypersensitivity (EMPATHY) study followed 300 premenarcheal adolescents (age M=11.29, SD=0.98 years) across the menarcheal transition with three visits comprising a comprehensive multimodal sensory testing battery: visual and auditory unpleasantness, a non-invasive bladder filling task, pressure pain thresholds, after-pain ratings, cold pain, and conditioned pain modulation. A principal component (PC) analysis of 24 baseline measures (n=214) identified three components accounting for 34.2% of variance: MMH (PC1; 16.5% of variance), a pressure-pain stimulus-response function (PC2; 9.3%), and bladder hypersensitivity (PC3; 8.4%). All experimental measures, and four self-report sensory sensitivity questionnaires projected as supplementary variables, loaded onto PC1, supporting its interpretation as MMH and replicating the component structure previously observed in adult women. Longitudinal trajectories of MMH (n=140) were modeled with linear mixed-effects regression with menstrual pain, pelvic pain, time, and their interactions as predictors, adjusting for pubertal staging. MMH decreased across the menarcheal transition (p=0.029). Pelvic pain (p=1.24x10-6), but not menstrual pain (p=0.205), was positively associated with MMH, independent of time and pubertal development. These findings establish MMH as a measurable, replicable sensory phenotype in early adolescence that covaries with non-menstrual pelvic pain, and position it as a candidate early marker of nociplastic pain risk. Future studies are needed to support interventions and screening for elevated sensory sensitivity before pain becomes clinically entrenched.

## Full Text Availability

The license terms selected by the author(s) for this preprint version do not permit archiving in PMC. The full text is available from the preprint server.

